# Endoscopic features of lymphoid follicles using blue laser imaging (BLI) endoscopy in the colorectum and its association with chronic bowel symptoms

**DOI:** 10.1371/journal.pone.0182224

**Published:** 2017-08-01

**Authors:** Tomomitsu Tahara, Kazuya Takahama, Sayumi Tahara, Dai Yoshida, Noriyuki Horiguchi, Tomohiko Kawamura, Masaaki Okubo, Mitsuo Nagasaka, Yoshihito Nakagawa, Makoto Urano, Tomoyuki Shibata, Tetsuya Tuskamoto, Hiro-o Ieda, Makoto Kuroda, Naoki Ohmiya

**Affiliations:** 1 Department of Gastroenterology, Fujita Health University School of Medicine, Toyoake, Japan; 2 Endoscopic Center, Ieda Hospital, Toyota, Japan; 3 Department of Diagnostic Pathology I, School of Medicine, Fujita Health University, Toyoake, Japan; University Hospital Llandough, UNITED KINGDOM

## Abstract

**Background/Aim:**

In the colorectum, lymphoid follicles hyperplasia (LH) is sometimes observed as small, round, yellowish-white nodules. The novel image-enhanced endoscopy system named blue laser imaging (BLI) provides enhanced the contrast of surface vessels using lasers for light illumination. We investigated the endoscopic features of LH observed by using BLI endoscopy and its association with chronic bowel symptoms.

**Patients/Methods:**

300 participants undergoing colonoscopy for various indications were enrolled. Entire colorectum was observed by using BLI-bright mode with non-magnification view. LH was defined as well demarcated white nodules. Elevated LH with erythema was distinguished as LH severe.

**Results:**

LHs were observed more clearly by using BLI-bright mode compared to conventional white light colonoscopy and were also histologically confirmed as intense infiltration of lymphocytes or plasmacytes. LH was observed in 134 subjects (44.6%) and 67 (22.3%) were LH severe. LH was associated younger age (Odds ratio (OR) = 1.05, 95%Confidence Interval (95%CI) = 1.03–1.07, *P*<0.0001) and chronic bowel symptoms including constipation, hard stools, diarrhea and loose stools (all LH: OR = 4.03, 95%CI = 2.36–6.89, *P*<0.0001, LH severe: OR = 5.31, 95%CI = 2.64–10.71, *P*<0.0001). LH severe was closely associated with both constipation associated symptoms (OR = 3.94, 95%CI = 1.79–8.66, *P* = 0.0007) and diarrhea associated symptoms (OR = 5.22, 95%CI = 2.09–13.05, *P* = 0.0004). In particular, LH severe in the ascending colon was strongly associated with bowel symptoms (*P*<0.0001).

**Conclusion:**

LH, visualized by using BLI endoscopy was associated with bowel symptom, raising the possibility of pathogenic role of this endoscopic finding in the functional lower gastrointestinal disorders.

## Introduction

Irritable bowel syndrome (IBS) is a widespread functional disorder in the lower gastrointestinal tract that is characterized by chronic bowel symptoms, including intermittent abdominal pain and altered bowel habits (diarrhea and/ or constipation) [1]. IBS is associated with impaired quality of life and reduced work productivity [2], and is an economic burden for both the patients and society [3–6].

Although the pathophysiology of IBS is not completely known, presence of colonic mucosal low-grade inflammation has been recognized as an important pathogenesis [7]. An increased number of intraepithelial inflammatory cells was found in patients with IBS [8–10], while the lymphoid follicles hyperplasia in the small and large intestine was associated with food hypersensitivity and bowel symptoms [11]. This raises the possibility that visualization of these inflammatory state as an endoscopic findings allow as to precise diagnosis of patients with chronic bowel symptoms such as IBS.

Many clinical studies have reported on the diagnostic performance of image enhanced endoscopy (IEE) techniques such as narrow-band imaging (NBI) for diagnosing endoscopic lesions [12–14]. NBI enhances the visualization of the surface vascular pattern using optical filters against a xenon lamp that allows narrow-band light to pass at wavelengths of 415 and 540nm. Combining the NBI and magnifying endoscopy provides accurate real-time diagnostic performance in inflammatory or neoplastic lesions in the gastro intestinal tract compared to the conventional white light [12,15]. Recently, Fujifilm developed an endoscope system with a semiconductor laser as a light source. The system includes two types of lasers with wavelengths of 410 and 450-nm. The 450-nm laser irradiates phosphor to produce illumination light similar to that obtained with a xenon lamp. The combination of strong 410-nm laser light, weak 450-nm laser light and fluorescent light enables blue laser imaging (BLI) via narrow-band light observation. Magnifying endoscopy with BLI is useful for evaluating mucosal surface information such as surface blood vessel and structure patterns [16–18].

In the colorectum, small, round, yellowish-white nodules, which is presumably an endoscopic feature of lymphoid follicles hyperplasia (LH), are sometimes observed in healthy subjects. It has been reported that LH in the gastric mucosa can be clearly visualized using NBI endoscopy [19]. BLI endoscopy provides enhanced the contrast of surface vessels using lasers for light illumination. Moreover, the BLI has bright mode, which achieves a brighter image than usual mode to maintain the enhanced contrast of surface vessels from a far field of view. The aim of the present study was to investigate the endoscopic features of LH observed by using BLI colonoscopy and its association with chronic bowel symptoms.

## Patients and methods

### Ethics statement

This study was approved by the Human Research Ethics Committee of the Fujita Health University School of Medicine. Each participant provided written informed consent for their clinical and laboratory data to be used and published for research purposes. The study was conducted according to the principles expressed in the Declaration of Helsinki.

### Patients

Study participants were prospectively enrolled from cancer-free patients of the endoscopy Center of Fujita Health University and Ieda Hospital from December 2014 to October 2015. Three hundred participants were initially invited, and all agreed to participate. All participants underwent total colonoscopy for various indications, including fecal immunological test positive, yearly follow up examination screening, concern about colonic diseases. Excluded from the study were patients who had malignancy, inflammatory bowel diseases, severe systemic disease, Celiac disease, acute infection including acute enteritis; those who had had a history of abdominal surgery. We also performed clinical tests for hepatitis B and C virus, treponema pallidum HIV before examination and confirmed that all participants were negative for all these tests. Fujita Health University School of Medicine approved the protocol, and written informed consent was obtained from all participants.

### Assessment of chronic bowel symptoms, endoscopic procedure, definition of LH

**Fig 1:**
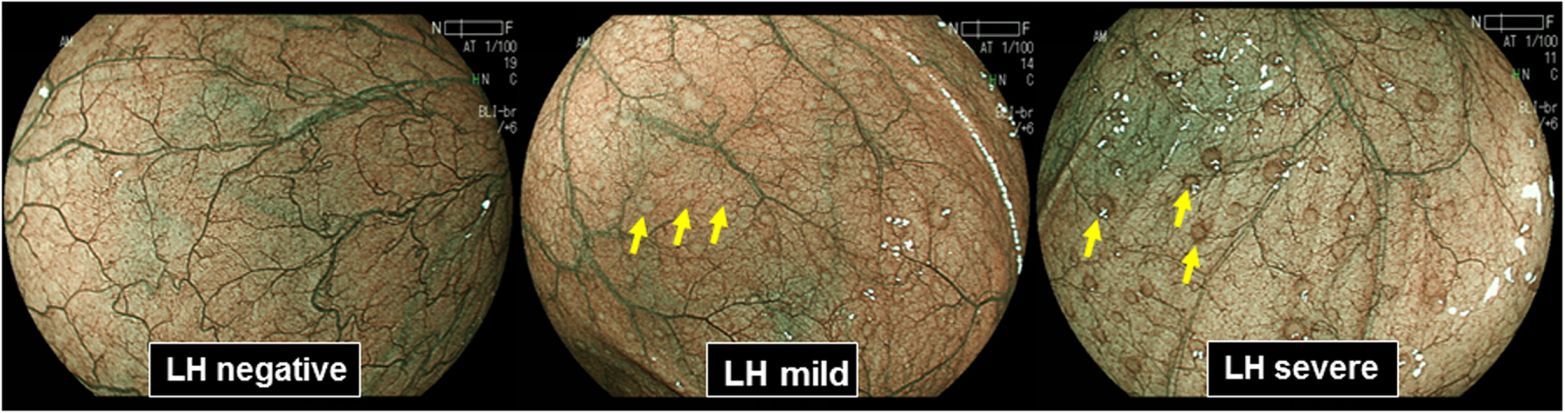
Representative endoscopic findings of lymphoid follicles hyperplasia (LH), observed by the BLI colonoscopy. LH was defined as well demarcated white nodules. Elevated LH with erythema was distinguished as LH high (right). All other LHs were considered as LH mild (center). Yellowish arrows indicates LHs.

Patients took 1.5 L polyethylene glycol solution (MOVIPREP; EA Pharma Co., Tokyo, Japan) on the morning before the examination to clean their entire colon. Before the colonoscopy, chronic bowel symptoms were assessed by the face to face questionnaire for all patients. Patients with chronic bowel symptoms were identified as having a primary compliant of either continuous or intermittent bowel symptoms including constipation, hard stools, diarrhea and loose stools at least for three month. Subjects who were negative for these bowel symptoms with in last 12 months were considered as asymptomatic subjects. The video endoscope used in this study was an EC-L590ZW (FUJIFILM Corporation, Tokyo, Japan). After inserting colonoscope into the cecum, BLI light source was turned on, and the entire colorectum was observed by the BLI-bright mode with non-magnification view. It has been reported that LH in the gastric mucosa can be clearly visualized as small, round, yellowish-white nodules using NBI endoscopy, which is another IEE endoscopic system [19]. According to this study, the presence of LH was defined as well demarcated white nodules. In particular, elevated LH with erythema was distinguished as LH severe (Fig 1). During the examination, at least 40 photographs were taken from the entire colorectum, even if there was no obvious lesion. If the obvious lesion such as polyps were seen, biopsy or polypectomy was performed. All of the endoscopic examinations were performed by a single expert endoscopist (T.T). Using the endoscopic photographs, presence of LH in each case was judged by the consensus manner among the three expert endoscopists (T.T, K.T and M.O).

### Histological examination

Among 16 patients who were judged as having LH severe, 22 targeted biopsies were taken from the LH. Histological examination was performed by the pathologist (S.T) using the Hematoxylin and Eosin stained slide.

### Visibility evaluation of LH using white light colonoscopy and BLI-bright mode

To compare the visibility of LH using conventional white light colonoscopy and the BLI-bright mode, paired endoscopic photographs using conventional white light colonoscopy and the BLI-bright mode were selected from the 88 patients who were considered as positive for LH. The selection of images were based on the best image quality as well as the exactly the same location where the paired endoscopic photographs were taken. All images were sorted by an endoscopist who were blinded to the patient’s characteristics. The chart numbers of the patients on the endoscopic images were all processed and masked and separated and arranged in random order for the evaluation of visibility of LH. The visibility of LH was scored from 0 (invisible), 1 (visible but unclear), 2 (visible) and 3 clearly (visible). This assessment was performed by the consensus manner of two independent endoscopists who are blinded about the patient’s characteristics.

### Data correction and statistical analysis

Categorical values among different groups were compared using the Chi Square Statistics.

Continuous values among two and three different groups were compared using the Student's t-test and the one-way ANOVA, respectively. The odds ratios (OR) and 95% confidence intervals (CI) were also calculated by the logistic regression with adjustment for age and gender. A P value of less than 0.05 was considered statistically significant.

## Results

### LH observed by BLI colonoscopy and its association with clinic-pathological features

Clinic-pathological features of three hundred subjects are shown in the Table 1. For all subjects, presence of LH was carefully evaluated throughout the entire colorectum using BLI-bright mode. In overall subjects, LH was observed in 134 subjects (44.6%). LHs can be also divided into two groups according to the presence of clear elevation and erythema: LH severe and LH mild (Fig 1). Among them, 67 (22.3%) were LH severe. Distribution of LHs in the colorectum was 25.3%, 37.7%, 15.3%, 14% 16.7%, and 15.7% in the appendix (cecum), ascending, transverse, descending, and sigmoid colons and rectum, respectively (S1 Fig).

**Table 1:** Clinic-pathological characteristics of 300 participants.

Variables	
*Median age (range)*	60 (22–84)
*Male/Female*	180 / 120 (60% / 40%)
*Reason for colonoscopy*	
Fecal occult blood positive	110 (36.7%)
Follow up examination	91 (30.3%)
Concern about colonic diseases	59 (19.7%)
Others	40 (13.3%)
*Chronic bowel symptoms*	
Asymptomatic	191 (63.7%)
Constipation symptoms	69 (23.0%)
Diarrhea symptoms	31 (10.3%)
Constipation + diarrhea symptoms	9 (3.0%)

To compare the visibility of LHs among the BLI-bright mode and the conventional white light colonoscopy, we compared the visibility score of LH among those two different light sources.

**Fig 2:**
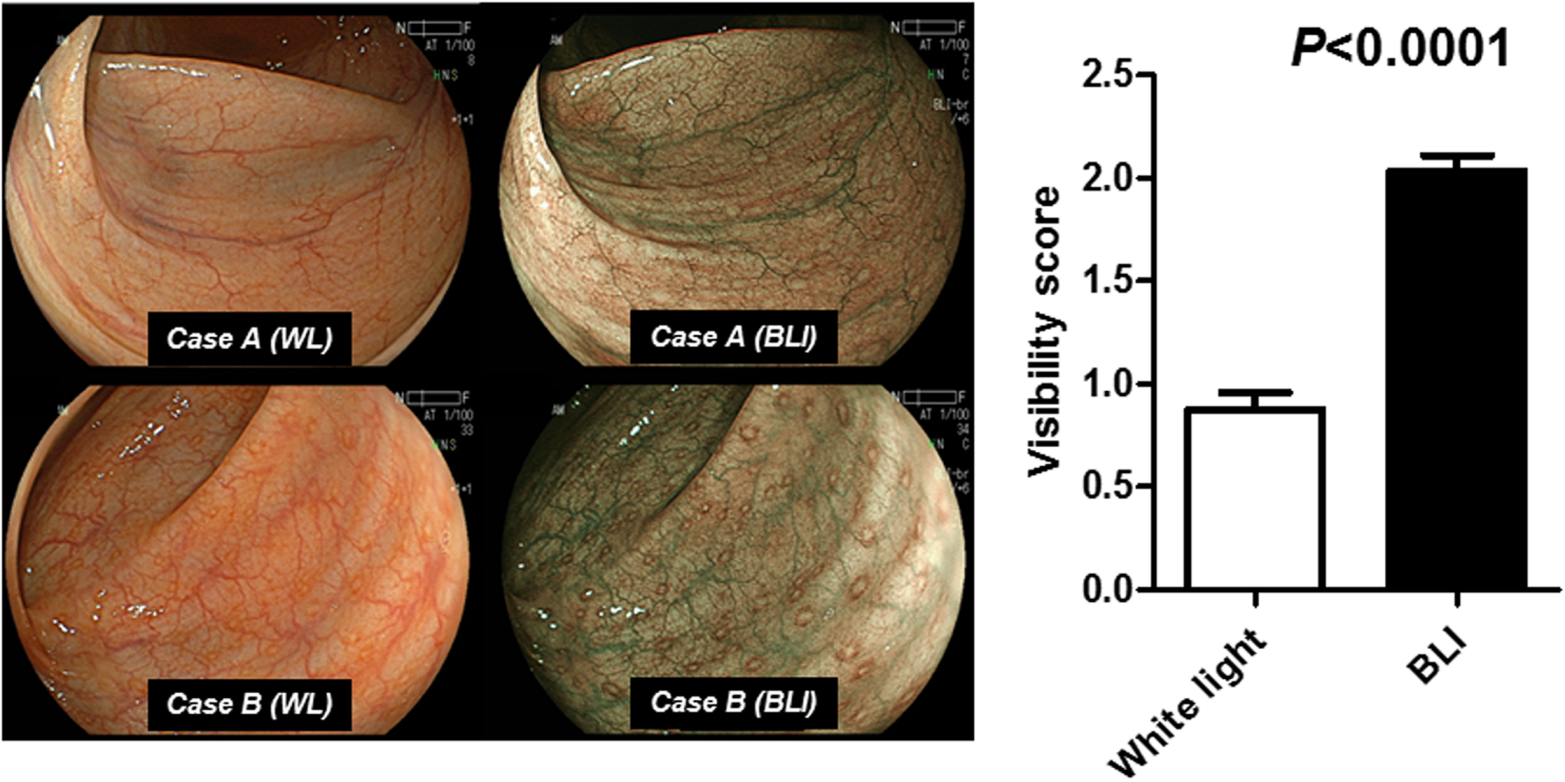
Left, lymphoid follicles hyperplasia (LH), observed by the conventional white light (WL) and BLI-bright mode (BLI) colonoscopy in two cases. Right, visibility score for detecting LH using the WL and the BLI colonoscopy. The statistical analysis was performed using the Student's t-test.

**Fig 3:**
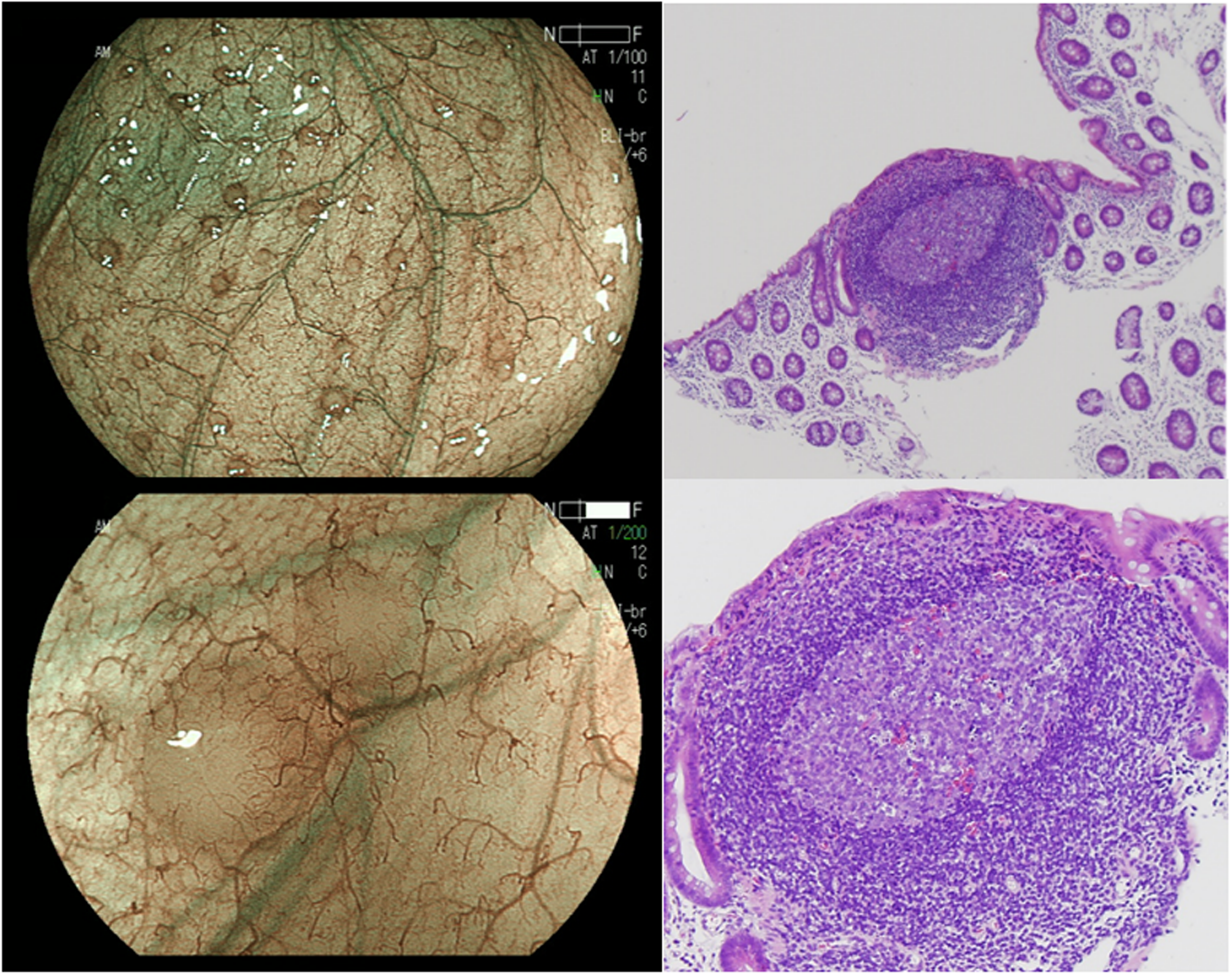
Histological finding of lymphoid follicles hyperplasia (LH). LH was histologically confirmed as intense infiltration of lymphocytes or plasmacytes (right).

Among the patients who were considered as positive for LH, the paired endoscopic photographs using conventional white light colonoscopy and the BLI-bright mode was available for 88 patients. Then we used these paired endoscopic photographs for this analysis. The visibility score of LH using the BLI-bright mode was significantly higher than that of conventional white light colonoscopy (P>0.0001), suggesting that LHs are observed clearly by using BLI-bright mode, compared to the conventional white light colonoscopy (Fig 2). It has been reported that LHs detected by the IEE endoscopy is associated with lymphoid follicles with lymphocyte infiltration or intense inflammatory cell infiltration [19]. Histological assessment of 22 targeted biopsies from 16 patients with LH severe also demonstrated that intense but localized infiltration of lymphocytes or plasmacytes in 20 out of 22 biopsies, while infiltration of inflammatory cells was not observed in the back ground colonic epithelium in these biopsies (Fig 3). We next investigated the association between presence of LH with age and gender. We showed that presence of LH, especially the LH severe was significantly associated with younger age (*P*<0.0001: S2 Fig). On the other hand, no association was found between the presence of LH and gender (*P*>0.1: S2 Fig).

### Association between presence of LH and chronic bowel symptoms

We investigated the association between presence of LH and chronic bowel symptoms.

**Fig 4:**
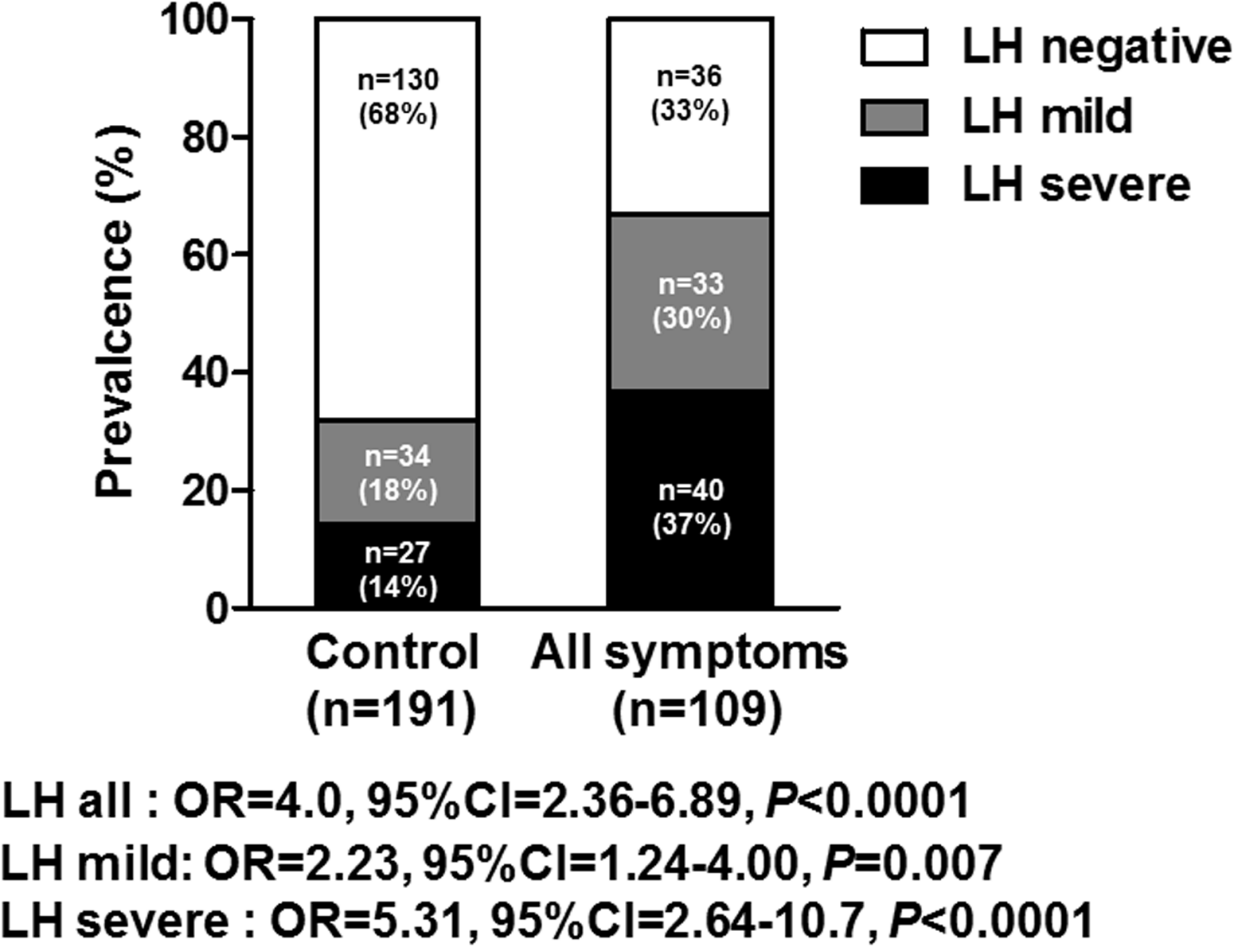
Association between lymphoid follicles hyperplasia and chronic bowel symptoms. Statistical analysis was performed using the logistic regression with adjustment for age, and gender.

**Fig 5:**
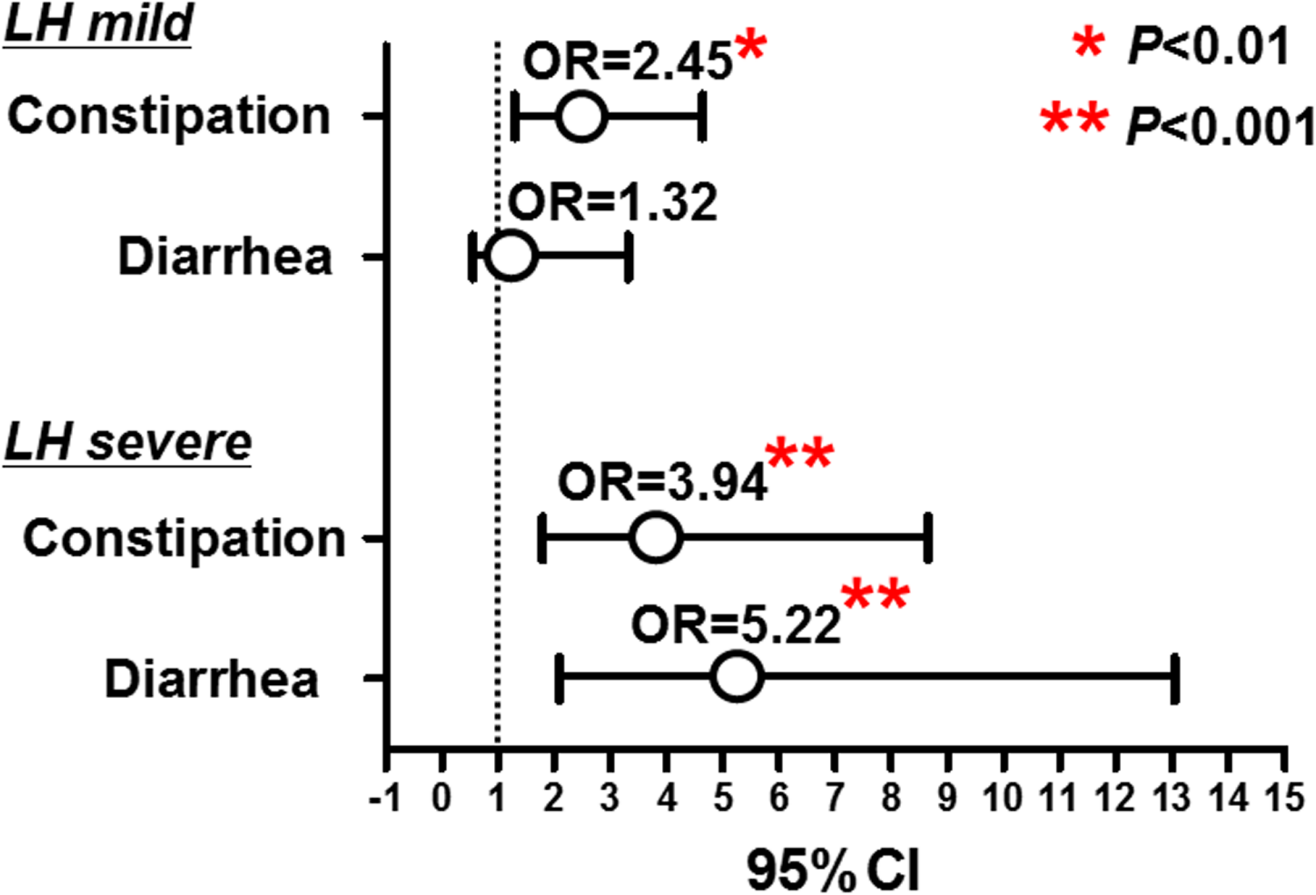
Association between lymphoid follicles hyperplasia (LH) and subtypes of chronic bowel symptoms. Statistical analysis was performed using the logistic regression with adjustment for age, and gender.

**Fig 6:**
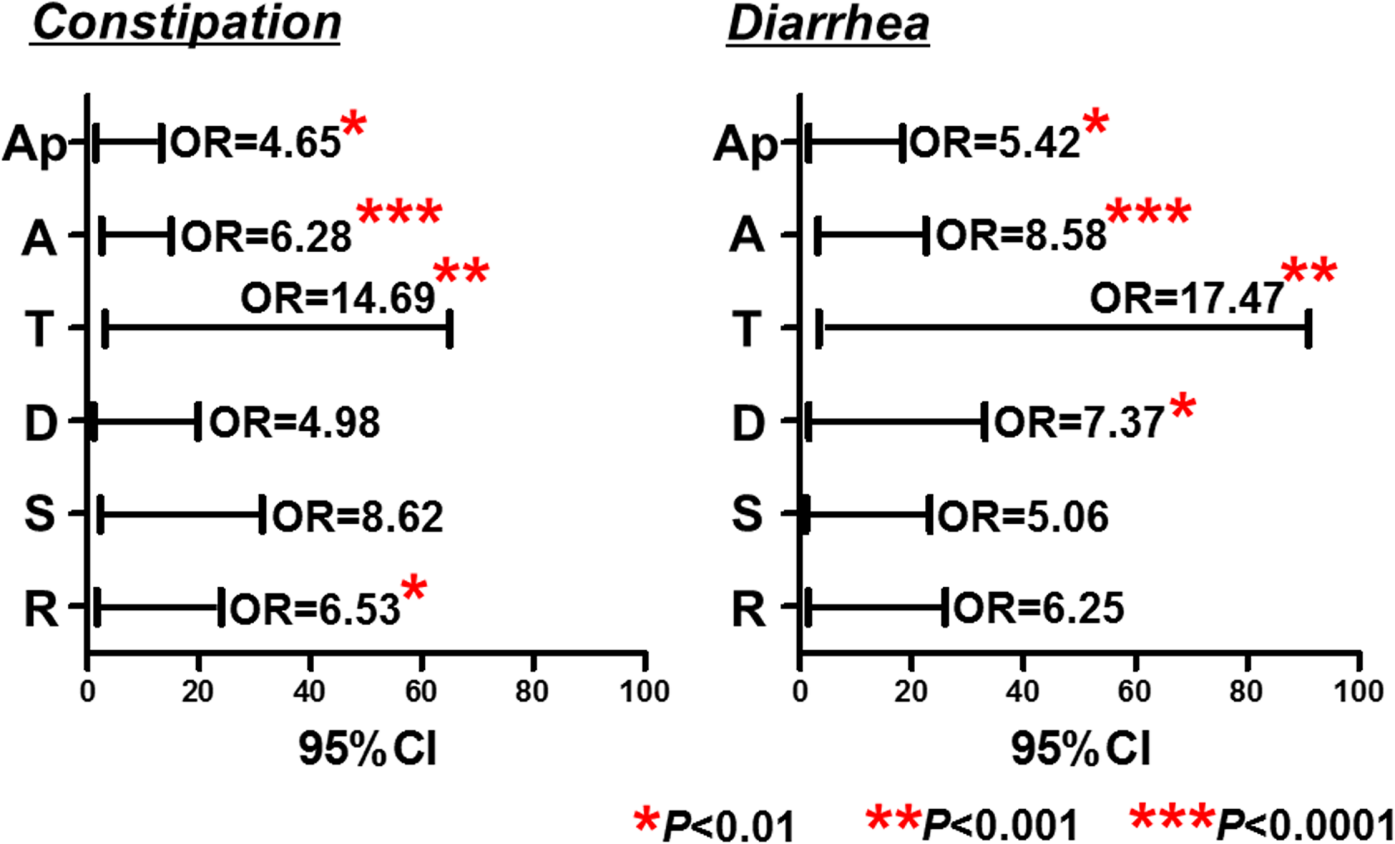
Association between the location of lymphoid follicles hyperplasia (LH) severe and chronic bowel symptoms. Statistical analysis was performed using the logistic regression with adjustment for age, and gender.

Chronic bowel symptoms were assessed by the face to face questionnaire for all patients for all three hundred subjects. Among all, 191 (63.7%) were asymptomatic, while 69 (23.0%) and 31 (10.3%) subjects were considered to have constipation and diarrhea related chronic bowel symptoms, respectively. In addition, 9 (3.0%) had both constipation and diarrhea related chronic bowel symptoms (Table 1). Prevalence of LH (LH mild plus severe) in the asymptomatic subjects was 32% (61/191), while the prevalence of LH in the subjects with chronic bowel symptoms were 67% (73/109). The logistic regression with adjustment for age, gender demonstrated that the presence of LH was significantly associated with chronic bowel symptoms (OR = 4.0, 95%CI = 2.36–6.89, *P*<0.0001: Fig 4). The sensitivity and the specificity of all LH (LH mild plus severe) for predicting chronic bowel symptoms was 67% and 68%, respectively. We also investigated whether the subtypes of LHs: LH severe and mild would be associated with chronic bowel symptoms including its subtypes. We showed that LH severe was more closely associated with chronic bowel symptoms compared to the LH mild (LH mild: adjusted OR = 2.23, 95%CI = 1.24–4.00, *P* = 0.007. LH severe: adjusted OR = 5.31, 95%CI = 2.64–10.71, *P*<0.0001: Fig 4). The closer association between LH severe was also confirmed when dividing subjects according its subtypes: constipation and diarrhea related symptoms (constipation associated symptoms: OR = 3.94, 95%CI = 1.79–8.66, *P*<0.001; diarrhea associated symptoms: OR = 5.22, 95%CI = 2.09–13.05, *P*<0.001) (Fig 5). We also investigated whether the distribution of LH in the colorectum would be associated with chronic bowel symptoms including its subtypes. Regarding the LH severe, which was more closely associated with chronic bowel symptoms, we showed that LH severe in the ascending colon was strongly associated with bowel symptoms including both constipation and diarrhea related symptoms (both *P*<0.0001: Fig 6).

## Discussion

LHs in the gastric mucosa can be clearly visualized using NBI endoscopy [19], while BLI has bright mode, which achieves a brighter image than usual mode to maintain the enhanced contrast of surface vessels from a far field of view. We compared visibility of LHs using the conventional white light colonoscopy and the BLI-bright mode. Although the comparison of white light and the BLI-bright mode was available in 88 patients with LH positive patients, the result suggest that LHs are clearly visualized by the BLI colonoscopy by the non-magnification view compared to the conventional white light colonoscopy. Recent study demonstrated that colorectal polyps are more easily visible with the BLI bright mode compared with compared to the conventional white light colonoscopy [20]. BLI colonoscopy with bright mode will be useful to screen tiny endoscopic lesions, such as LHs, without magnification view.

Since the LHs in the small and large intestine was associated with food hypersensitivity and bowel symptoms [11], we investigated the clinical relevance of LHs visualized by the BLI colonoscopy. We showed that LH was associated younger age and chronic bowel symptoms including constipation and diarrhea related symptoms. Although the presence of colonic mucosal low-grade inflammation has been recognized as an important pathogenesis in IBS, direct link between the chronic bowel symptoms and endoscopically visualized LHs has not been clearly reported, probably due to the lack of sensitivity to detect LHs by the conventional white light colonoscopy. We showed that LHs are commonly observed in asymptomatic subjects (32%) but more frequent in patients with chronic bowel symptoms (67%). Our result provided the evidence that presence of LHs using the BLI colonoscopy is useful endoscopic appearance to predict chronic bowel symptoms. Presence of LH has been correlated with food hypersensitivity and bowel symptoms especially in the younger generations [11]. Our association between LHs with younger age and chronic bowel symptoms may suggest that LHs decrease by age, while patients who remain LHs in the adulthood may develop chronic bowel symptoms. On the other hand, it would be usual that the younger patients don’t typically undergo screening colonoscopy unless they have symptoms. Although the association between LH chronic bowel symptoms was confirmed by the logistic regression with adjustment for age, it needs to be investigated the prevalence of LHs in the larger cohort of younger patients.

An increased number of intraepithelial lymphocytes has been found in patients with IBS [8,9], while other studies showed that mast cells infiltration was increased in IBS especially who have past history of acute infection [10]. Histological assessment of LH showed intense infiltration of lymphocytes or plasmacytes, suggesting that BLI endoscopy can visualize colonic mucosal low-grade inflammation that is associated with chronic bowel symptoms. We could not obtain detailed clinical information regarding the past history of acute infection in patients with bowel symptoms. Further study will be needed to characterize the differences of LHs in IBS with or without history of acute infection by using the BLI colonoscopy.

We investigated whether the different types of LHs including their appearances and locations would be associated with chronic bowel symptoms. We showed that LH severe, characterized as elevated LH with erythema was more closely associated with chronic bowel symptoms including both the constipation and diarrhea related symptoms. Moreover, LH severe in the ascending colon was strongly associated with chronic bowel symptoms including their subtypes. This indicates the importance of discriminating the appearances or locations of LHs to predict chronic bowel symptoms. Our data suggest that LH severe may reflect severe degree of inflammatory response or inflammation that may be closely associated with chronic bowel symptoms. As for the subtypes of symptoms, LH severe was significantly associated with both different subtypes of bowel symptoms. However, patients with diarrhea related symptoms in our data set was less frequent (10.3%) compared to the constipation related symptoms (23.0%), suggesting that the association between LH severe with diarrhea related symptoms might be stronger than that with constipation in view of statistical power. Still, why the LH is associated with different subtypes of chronic bowel symptoms needs to be clarified. This is a preliminary data reporting the potential association between LH and chronic bowel symptoms, but our findings raises the possibility of pathogenic role of this endoscopic finding in the functional lower gastrointestinal disorders, opening the avenue of research on IEE techniques in this field. We believe that our result provide salient findings despite it is a preliminary investigation.

## Supporting information

S1 Fig Distribution of lymphoid follicles hyperplasia (LH) in the colorectum. Ap, appendix (cecum); A, ascending colon; T, transverse colon; D, descending colon; S, sigmoid colon; R, rectum.
(TIF)

S2 Fig Association between lymphoid follicles hyperplasia (LH), age (left) and gender (right). (TIF)
