# Supplementary figures and images for: Endoscopic features of lymphoid follicles using blue laser imaging (BLI) endoscopy in the colorectum and its association with chronic bowel symptoms
Source: PLoS One. 2017 Aug 1;12(8):e0182224. doi: 10.1371/journal.pone.0182224 (PMC5538676; doi:10.1371/journal.pone.0182224)

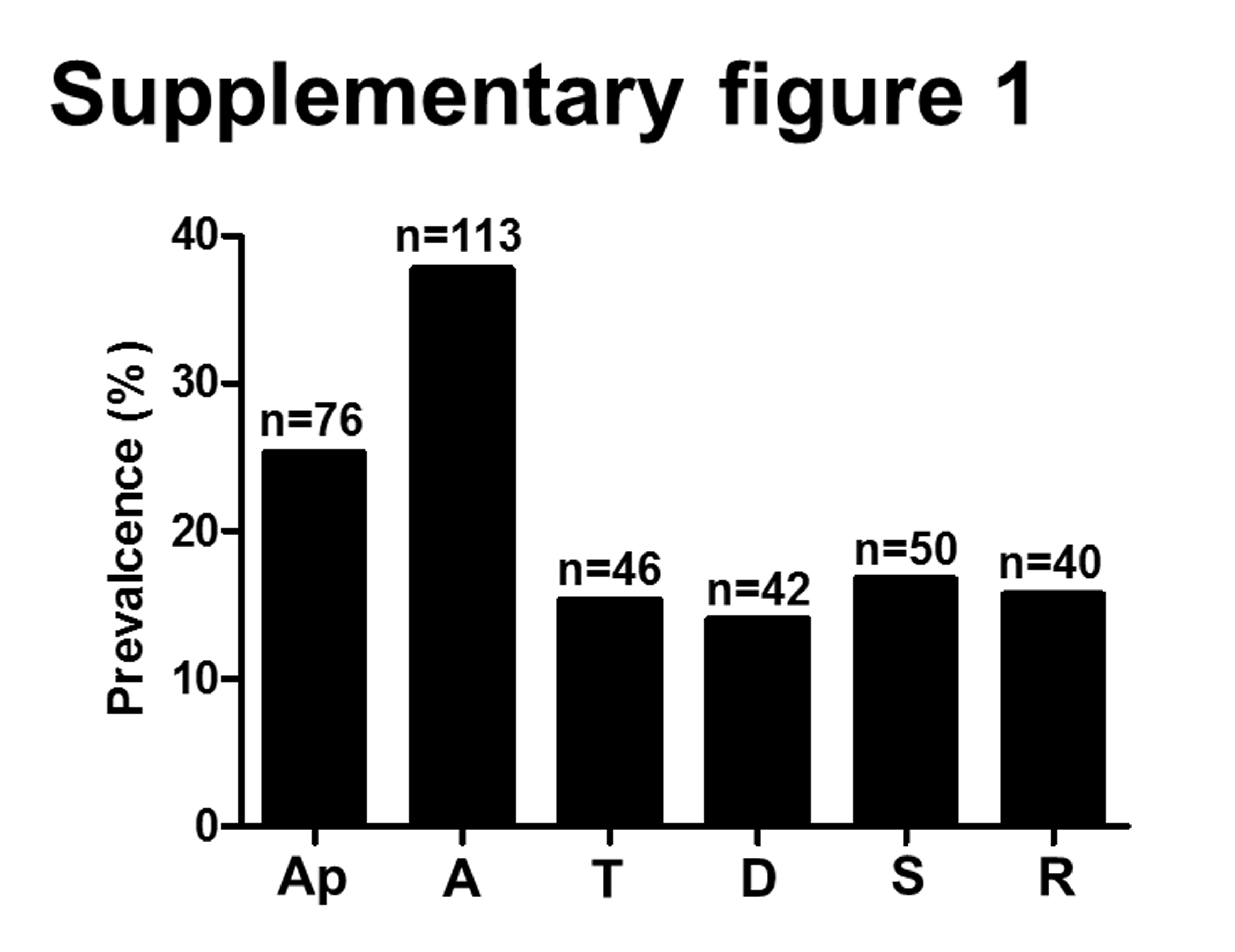

Supplement: S1 Fig — Ap, appendix (cecum); A, ascending colon; T, transverse colon; D, descending colon; S, sigmoid colon; R, rectum. (TIF) [file pone.0182224.s001.TIF]

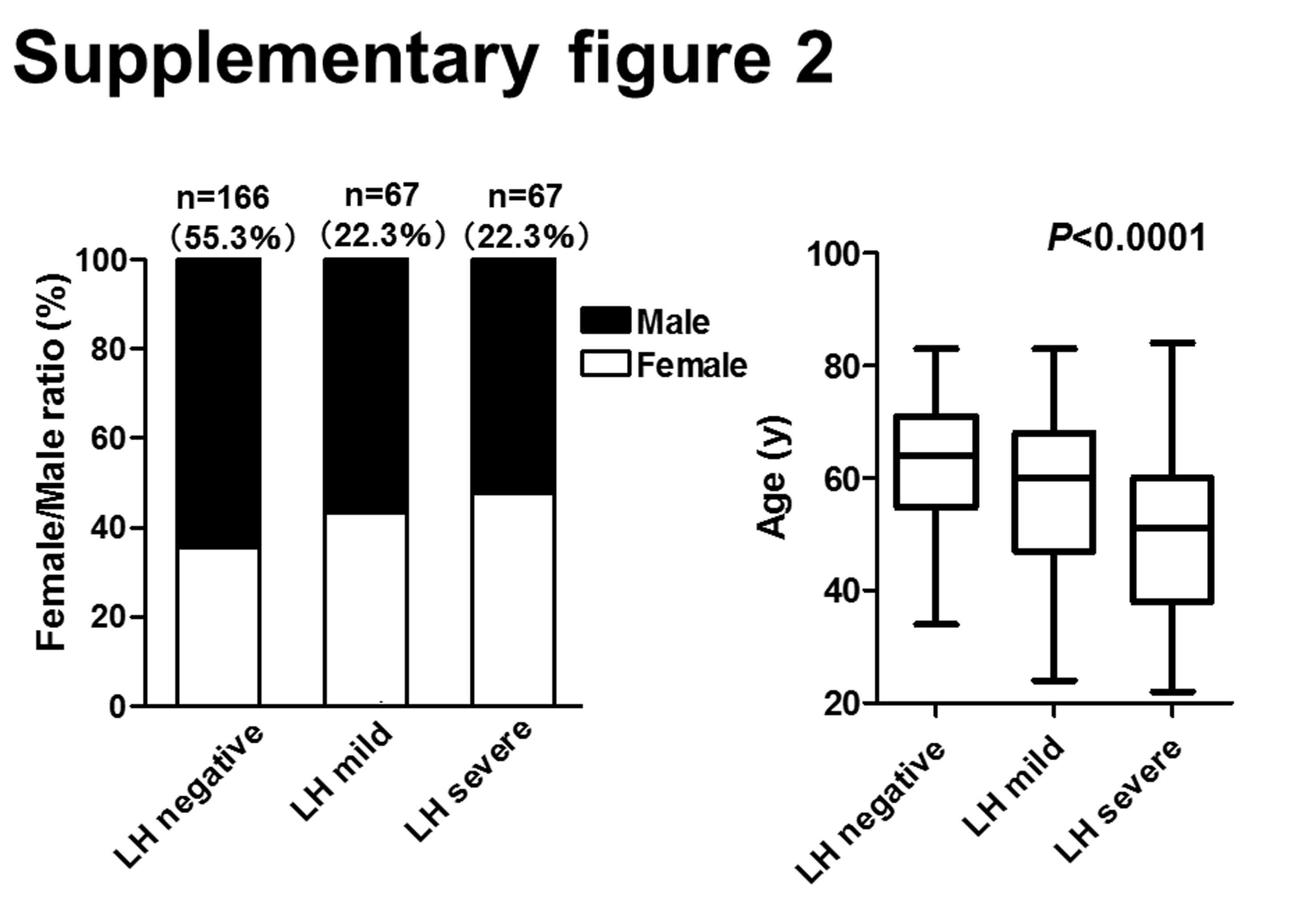

Supplement: S2 Fig — (TIF) [file pone.0182224.s002.TIF]
